# Technology-assisted assessment of spasticity: a systematic review

**DOI:** 10.1186/s12984-022-01115-2

**Published:** 2022-12-09

**Authors:** Xinliang Guo, Rebecca Wallace, Ying Tan, Denny Oetomo, Marlena Klaic, Vincent Crocher

**Affiliations:** 1 UoM and Fourier Intelligence Joint Robotics Laboratory, Mechanical Engineering Department, The University of Melbourne, Melbourne, Australia; 2 Allied Health Department, The Royal Melbourne Hospital, Melbourne, Australia; 3 School of Health Sciences, The University of Melbourne, Melbourne, Australia

**Keywords:** Spasticity, Outcome measure, Neuro-rehabilitation, Neurological disorders, Muscle tone

## Abstract

**Background:**

Spasticity is defined as “a motor disorder characterised by a velocity dependent increase in tonic stretch reflexes (muscle tone) with exaggerated tendon jerks”. It is a highly prevalent condition following stroke and other neurological conditions. Clinical assessment of spasticity relies predominantly on manual, non-instrumented, clinical scales. Technology based solutions have been developed in the last decades to offer more specific, sensitive and accurate alternatives but no consensus exists on these different approaches.

**Method:**

A systematic review of literature of technology-based methods aiming at the assessment of spasticity was performed. The approaches taken in the studies were classified based on the method used as well as their outcome measures. The psychometric properties and usability of the methods and outcome measures reported were evaluated.

**Results:**

124 studies were included in the analysis. 78 different outcome measures were identified, among which seven were used in more than 10 different studies each. The different methods rely on a wide range of different equipment (from robotic systems to simple goniometers) affecting their cost and usability. Studies equivalently applied to the lower and upper limbs (48% and 52%, respectively). A majority of studies applied to a stroke population (N = 79). More than half the papers did not report thoroughly the psychometric properties of the measures. Analysis identified that only 54 studies used measures specific to spasticity. Repeatability and discriminant validity were found to be of good quality in respectively 25 and 42 studies but were most often not evaluated (N = 95 and N = 78). Clinical validity was commonly assessed only against clinical scales (N = 33). Sensitivity of the measure was assessed in only three studies.

**Conclusion:**

The development of a large diversity of assessment approaches appears to be done at the expense of their careful evaluation. Still, among the well validated approaches, the ones based on manual stretching and measuring a muscle activity reaction and the ones leveraging controlled stretches while isolating the stretch-reflex torque component appear as the two promising practical alternatives to clinical scales. These methods should be further evaluated, including on their sensitivity, to fully inform on their potential.

**Supplementary Information:**

The online version contains supplementary material available at 10.1186/s12984-022-01115-2.

## Background

The definition of spasticity has long been debated in published studies and amongst clinicians. This definition has sometime encompassed any increase in muscle tone of various physiological origins, whether they are constant (and then referred to simply as “tone”, “hyper-resistance” or “hyper-tonicity”) or are only velocity-dependent (and in which case are due to an exaggerated stretch-reflex) [1]. Still, the more commonly used definition—adopted in this work—remains the more specific one proposed by Lance in 1980: “a motor disorder characterised by a velocity dependent increase in tonic stretch reflexes (muscle tone) with exaggerated tendon jerks, resulting from hyperexcitability of the stretch reflex, as one component of the upper motor neuron syndrome” [2]. This definition has been recently confirmed and updated by a European consensus, stating that “spasticity refers to velocity dependent stretch hyperreflexia as part of hyper-resistance” [3]. These definitions should still be taken with care when considering the measurement modality and procedure used. Indeed, Lance’s definition was primarily derived from muscle activity observations, while today clinical practices rely on the measure of an exaggerated force or torque response (e.g. a catch angle). In addition, a continuous velocity-dependent torque response has also been recently demonstrated for the elbow joint by McPherson et al. [4]. Overall, past the phenomenon definition, it remains unclear as to which modality is appropriate to characterise spastic responses.

### Significance of spasticity and its assessment

Spasticity is a highly prevalent symptom in people suffering a neurological injury, with estimates ranging from 30 to 80% after stroke [5]. Upper limb spasticity following a stroke affects a large number of individuals in the chronic phase [6] and is strongly correlated with post-stroke pain [7] and limitation of patient’s engagement in rehabilitation [8, 9]. The socioeconomic burden for those with post-stroke spasticity is estimated to be four times greater than for stroke survivors without spasticity [10]. Therefore, effective management of post-stroke spasticity remains a critical issue of importance in the field of neurological rehabilitation [11]. However, measuring effectiveness of treatments requires sensitive, valid and reliable assessment tools.

The Modified Ashworth Scale (MAS) and Modified Tardieu Scale (MTS) are the more commonly used measures of spasticity in clinical practice [12]. These measures have important limitations, especially the limited ability to distinguish between spasticity—velocity dependent and of neural origin, as per Lance’s definition—on one hand and tone or stiffness—of non-neural origin—on the other hand. The importance of this differentiation has been recently stressed by a European consensus [3].

Specifically, the MAS rates the reaction of the assessed muscle to stretch using a six point scale [13]. The measure evaluates the resistance torque at a single, approximately defined stretching velocity, so it cannot capture the velocity-dependent component of spasticity. The MAS has also been shown to have only moderate intra-rater and inter-rater reliabilities, leading to questions regarding the overall validity of this tool in the measurement of spasticity [14]. The MTS [15] has been recommended as a more appropriate measurement of spasticity [16]. Like the MAS, this tool rates the reaction of the affected muscle using a Likert scale from 0—no resistance to 4—unfatigable clonus. The primary difference between the two measures is that the MTS explicitly considers velocity-dependent characteristics by requiring the clinicians to stretch the joint at two different velocities, “as slow as possible” and “as fast as possible” [17]. However, the MTS does not fully reflect the variation of the intensity of the stretch induced by the velocity as the scale is only based on the angles at which the muscle reaction occurs. Its sensitivity is also limited by the ability of the rater to evaluate the reflex torques accurately and its inter-rater reliability is dependent on the experience of the clinician [15].

### Technology assisted assessments

Given the importance of spasticity evaluation and its relevance to motor impairment and rehabilitation, together with the stated limitations of the existing clinical scales, many attempts have been made to offer efficient and reliable technological solutions to this evaluation. Two main classes of systems have been developed since the late 1980s [18, 19]: passive instruments, where the goal is to accurately measure the resistance force and/or muscle activity at a given joint which is manually stretched by a clinician; and active (i.e. robotic) devices which produce a controlled movement of a specific joint at several possible velocities while measuring the resistance force or muscle activity.

These techniques use a variety of different apparatus and propose a variety of different outcome measures but have often been individually evaluated, for different populations, different joints and often relatively low number of subjects, making it challenging to define and compare their clinical relevance. Many of these measures have not been adopted into clinical practice, possibly due to the complexity of their apparatus, amongst other factors. Indeed, studies have found that perceived ease of use and perceived usefulness are strong predictors of clinician likelihood to adopt such devices in practice [20]. It is to note that despite the aforementioned limitations, the MAS and MTS are simple and quick assessments to administer, potentially explaining their predominance against instrumented measures with lower usability.

Although two recent dedicated reviews [21, 22] investigated robotic-assisted methods for spasticity, the restriction of their scope to robotic systems does not allow for a full picture and comparison of existing methods. A more complete picture of the field is provided in a review of systematic reviews encompassing all assessment methods [23]. This review shows the overall limited evaluation of the existing assessments but does not propose a specific categorisation—and thus comparison—of the methods used. Additionally, none of these previous reviews address the question of the usability of the assessment methods which is a critical point for clinical adoption, especially in comparison to the widely used existing clinical scales which have the benefit of being cost-effective and easy to administer.

This systematic review thus proposes to identify existing technology-assisted methods aiming to assess the level of spasticity. A classification based on the method characteristics is then proposed, allowing for a usability comparison. Finally the psychometric properties of the different outcome measures are analysed. The review scope encompasses any limb and joint (or muscle) and any condition leading to spasticity, as the underlying mechanism of spasticity and its manifestation are expected to remain consistent across these conditions.

## Methodology

### Search and screening

A systematic literature review search was performed on the Medline, Embase and IEEEXplore databases. The search query was constructed to identify papers of which title or abstract contain at least one keyword of each of the three following groups: (1) spasticity, (2) assessment and (3) technology. The keywords of each group were defined as follows:

1. spastic* (spastic, spasticity), muscle tone, muscular tone, hyperton* (hypertonia, hypertonic, hypertonicity);
2. assess* (assess, assessment), measure* (measure, measurement), quanti* (quantify, quantification, quantitative);
3. technolog* (technology, technological), instrument* (instrument, instrumental, instrumented), mechatronic, mechanical, muscle activity measurement, electromyography, EMG, sEMG, inertial measurement unit, IMU, force sensor, dynamometer, ergometer, robot* (robot, robotic, robotics), kinematic* (kinematic, kinematics, kinematical).

Note that the key terms of group (1) deliberately included terms that may not be specific to spasticity as per Lance’s definition. These terms were included to ensure to not exclude valid studies using an inappropriate terminology. The construct validity of each measure was then evaluated in a second time, as explained below. The technology group (3) was constructed to include any mechatronic and/or sensor based systems.

The search was restricted to papers published after January 2000 to exclude older results leveraging outdated technology. Both journal articles and full-text conference proceedings written in English were included. Additional papers identified outside of the search were also included.

Eligibility was assessed based on the paper abstract to ensure that the reported study was specific to spasticity, or more generally to muscle tone, and applied to a neurologically injured population. Only papers directly aiming at the assessment of spasticity were considered. As such papers only reporting spasticity treatments or management methods were not included. Finally, it was ensured that the papers were using or proposing a technology-assisted measure. Typically, studies assessing psychometric properties of non-instrumented clinical measures (such as MAS or MTS) were excluded. Abstracts of identified papers were then screened independently by two reviewers (XG and RW) for eligibility. In case of disagreement, inclusion decision was made by a third reviewer (VC).

The PRISMA methodology [24] was used to report the literature review.

### Data extraction and analysis

The full texts of the included papers were then analysed. The first objective was to characterise the spasticity assessment method used (or proposed). This step consisted of identifying the type of sensor(s) and device(s) used, the type of physiological measure(s), procedure, outcome measure and joint being assessed. When multiple outcome measures were proposed in the same study, only the one(s) claimed to be specific to spasticity by the authors were reported. When a paper presented several distinct assessment methodologies, these were considered independently. Conversely, when several papers were relative to the same assessment method, those were reported together.

The information extracted was used to populate a first table and further used to provide a full picture of technology-assisted assessments of spasticity.

The second step aimed at extracting, for each study, the relevant psychometric properties of the assessment and other information relative to its evaluation with the targeted population. When a study used more than one method, those were considered independently. Methods only tested with non-neurologically impaired populations were not considered at this stage. Specifically, information were sought regarding:

- the targeted population;
- the spasticity severity of the targeted population (in terms of a clinical score);
- the sample size, assessed here as number of limbs tested and either belonging to the test population or control (including own control);

and on the reporting of the following five psychometric properties.

1. The construct validity, evaluating the specificity of the measure based on Lance’s and the European consensus definitions. Two aspects were sought for evaluation: (1) is the measure (and/or procedure) accounting for the velocity dependent aspect of the phenomenon (independently of the type of the outcome measure); and (2) is the measure (and/or procedure) attempting to isolate the stretch reflex from any voluntary muscle component and other joint passive resistance? This was thus rated from 0 to 2.
2. The discriminant validity, based on the existence of a control group/limb and ability of the measure to discriminate between these groups. This was rated as Significant, Conditionally Significant (under specific conditions) or Non-Significant.
3. The clinical (concurrent) validity, based on its correlation with the clinical measures reported as a Kappa, Spearman or Pearson correlation coefficients and rated from Very Weak to Very Strong [25, 26].
4. The reliability, based on a rating of the repeatability from Poor to Excellent (ICC [27]).
5. The sensitivity evaluation, reporting the Minimal Detectable Change (MDC) or similar measures.

The information, when available, was used to populate a second extraction table. This extraction was performed by one of the authors (XG) and discussed among all the authors in case of doubt.

Due to the heterogeneity of the data, no quality appraisal of the studies was performed but this information was further used to analyse how the different assessment methods—and outcome measure(s)—have been investigated along the different psychometric properties.

For the assessment methods with a construct validity of two out of two (thus specifically evaluating spasticity as a velocity-dependent increase of the stretch reflex) a usability evaluation was performed. The administration time, equipment cost and portability of the equipment necessary to these methods were estimated. The administration time was estimated by the authors based on the procedure description, the required instrumentation (such as EMG sensors placement or exoskeleton adjustment) and the number of movements/actions required (see Appendix, Table 5). The administration time was then classified as either:

- comparable administration time to a MAS or MTS: less than 10 min;
- equivalent to a typical intervention session: 10 to 30 min;
- length of an extended session: 30 to 60 min or;
- longer than an extended session: more than 60 min.

The equipment cost was estimated using the cost of a standard equivalent equipment (see Appendix, Table 6) and classified as either:

- a disposable expense: less than $1000 USD;
- an expense requiring a departmental funding: $1000 to $10,000 USD;
- an expense requiring an institutional funding: $10,000 to $50,000 USD or;
- an expense requiring a grant or special funding: more than $50,000 USD.

Portability was estimated based on the less portable piece of equipment and classified as either:

- easily transportable (e.g. EMG sensors);
- transportable from room-to-room (e.g. Ultra-Sound system on wheels) or;
- not movable (e.g. BIODEX system).

## Results

**Fig. 1:**
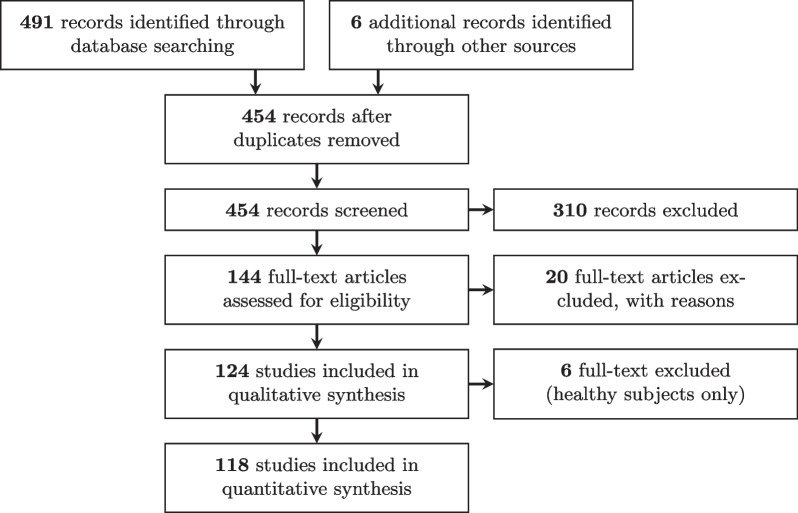
PRISMA diagram of the literature review

The search conducted in May 2021 identified 491 papers and six were added from other sources by the authors (see Fig. 1), 310 papers were excluded based on their abstract and 20 additional ones were excluded after a full-text review, leading to a total of 124 papers included in the analysis. During the screening phase, there was an agreement among the two reviewers on 384 papers whereas 70 required an arbitration.

### Available assessment methods

In total, 120 different assessments were identified. The extraction table summarising the assessment methodologies presented in each paper is available as a supplementary material (Additional file 1).

**Fig. 2:**
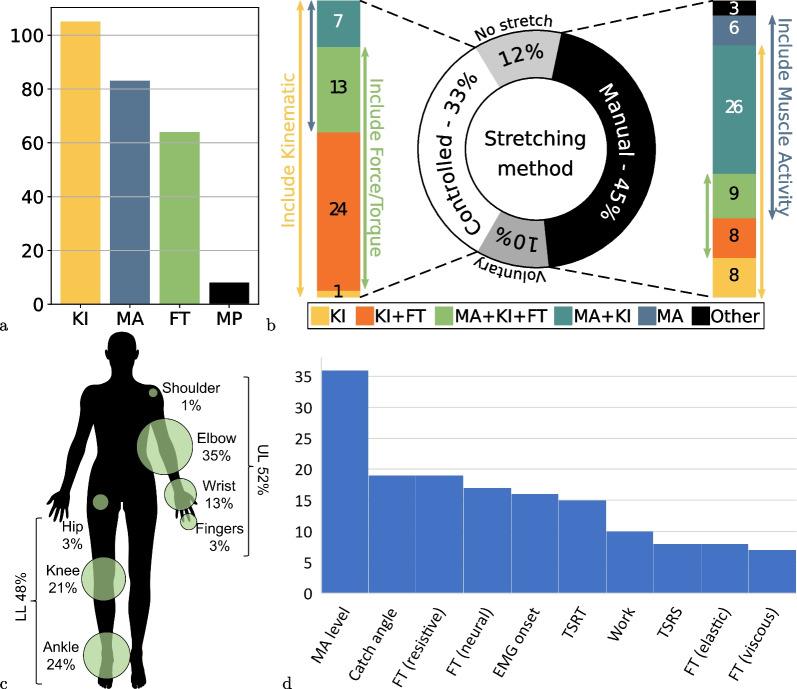
Distributions of studies by **a** types of measurement (some studies use more than one type of measurement); **b** types of stretch used and breakdown of measurements for the two main stretch categories; **c** joint(s) on which the methods have been applied to; and **d** most commonly encountered outcome measures (some studies have several outcome measures). *KI* kinematic measure, *MA* muscle activity measure, *FT* force/torque measure, *MP* muscle property measure

#### Physiological measures

Four categories of physiological measures—and their combinations—used to produce the outcome measure were identified. They are summarised in Table 1.

**Table 1:** Definition of the physiological measures categories

Abbr.	Denotations	Definitions
KI	Kinematic	A measure of the limb movement, either position or velocity
MA	Muscle Activity	A measure proportional to the muscle contraction intensity (e.g. EMG measurement)
FT	Force/Torque	A measure of the force exerted by the limb, or equivalent torque at a joint, to resist a limb movement
MP	Muscle Property	A measure of the mechanical condition (e.g. stiffness) of a muscle

A majority of studies used Kinematic measures (KI, N = 105), then Muscle Activity measures (MA, N = 83), then Force/Torque (FT, N = 64), and a few studies used intrinsic Muscle Properties (MP, N = 8) (see Fig. 2a). The most common combinations were Kinematic with Muscle Activity (N = 63), and Kinematic with Force/Torque (N = 56).

#### Stretching methods

Studies were further categorised based on the type of movement used defined in Table 2.

**Table 2:** Definition of the stretching methods categories

Abbr.	Denotations	Definitions
M	Manual Stretch	The subject’s limb is manually stretched by a practitioner
C	Controlled Stretch	The subject’s limb is stretched by a mechatronic device controlling the movement
V	Voluntary Stretch	The subject voluntarily controls their limb movement without external intervention
NS	No Stretch	The measure is performed at static pose(s) of the subject’s limb

Most studies relied either on Manual stretching of the limb (N = 60) or on Controlled stretching movements (N = 45). Only a small number of studies (N = 14) relied on Voluntary movements. This last option has the disadvantage of not providing a standard movement velocity—and its variations—but has the advantage of being more directly representative of the spasticity effect on patients function.

These stretches were applied at several different velocities in 69 studies—either in a randomised or increasing velocity order (N = 48 and N = 21 respectively)—which demonstrate how most methods tackle the velocity dependence aspect of spasticity. Still, in 44 studies, only one stretching velocity was used and 23 did not clearly report the number of velocities used or did not use any stretching (NS).

#### Joints of application

The different methods were equally applied to the upper or lower limb joints (52% vs 48%) but much more frequently to the more distal joints with only two studies relative to the shoulder and four to the hip (see Fig. 2c). Only four studies were applied to the fingers joints.

#### Devices used

Ten categories of technological devices could be identified in the different studies. Most of the studies used two or more types of devices. Fifty studies relied on active systems, either a robotic end-effector system (REE, N = 34) or a robotic exoskeleton (REXO, N = 16), among which they were coupled with EMG measurements in 23 cases. Electrical stimulation (STI) was used in four studies. Passive orthoses (ORT) were used to guide or stabilise the movement and measure either kinematic or kinetic data in 20 cases.

Purely in terms of measurement devices, EMG is the most commonly used system (N = 83), followed by goniometers (GON, N = 26) and IMUs (N = 17), dynamometers (DYN, N = 8) and finally Ultra-Sound or mechanomyography (US or MMG, N = 7).

#### Outcome measures

Seventy-eight different outcome measures were identified with only a few recurrent ones and multiple studies reporting several outcome measures. The largest category encompasses the Force/Torque level outcomes (N = 51), either resistive torques measured in varying conditions or the Force/Torque evolution over stretching angle or velocity. Thirty-six studies reported a Muscle Activity level, 19 reported a catch angle, 16 the presence of an EMG onset and 15 the Tonic Stretch Reflex Threshold (TSRT), sometimes with the associated Tonic Stretch Reflex Slope (TSRS). Figure 2d presents the most commonly encountered measures and a full list is available in the Additional file 1. Among the variety of other outcome measures reported, it is noted that six studies aimed at estimating a MAS score equivalent, either by reproducing the MAS procedure using technological equipment or by using machine learning techniques on a set of recorded features.

#### Types of measurement

When comparing the main stretching categories (Fig. 2b), not surprisingly, all methods using Controlled stretching relied on Kinematic measures—as it is directly provided and controlled by the stretching system. Quite naturally, Controlled methods also more commonly relied on Force/Torque measurements than Manual methods, as this measure can be directly provided by the mechatronics system. Instead, Manual methods tend to use Muscle Activity measures more frequently as an alternative to Force/Torque.

### Psychometric properties

The detailed data extraction table with the characteristics of each study, and for each outcome measure, is provided as a Supplementary material (Additional file 2). Six studies [28–33] were excluded from the psychometric properties analysis as they only recruited healthy subjects.

There is quite a large variety of study designs, which do not all aim at formally assessing the psychometric properties of the used—or proposed—assessment methods. As such, not surprisingly, no study reported all the expected items and six studies reported four of the five properties [34–39]. When psychometric items were reported, they also were commonly evaluated only for some of the outcome measures proposed.

Overall, more than half of the studies (N = 76) proposed or evaluated a method which scores less than two on the construct validity criterion, showing that it is either not velocity-dependent or does not attempt to isolate the stretch reflex component.

For 42 studies, at least one outcome measure was able to discriminate between the test and control populations, whereas this discrimination was not possible, or only under specific conditions, in 10 studies. 78 studies did not report any discriminant validity evaluation.

A Strong or Very Strong correlation of the evaluated measure with clinical measures of spasticity was found in 28 studies, out of the 62 reporting such evaluation. It is to note that in most of these studies (N = 33) the concurrent validity was evaluated against the MAS. Given the limited properties and limited specificity of the MAS, this raises the question of relevance of these correlations.

The repeatability of the proposed measures was reported in only 29 cases and was found excellent in 25 cases.

Sensitivity was evaluated in only three studies, either using a Minimal Detectable Change (MDC), a Smallest Real Difference (SRD) or a Smallest Detectable Difference (SDD).

The targeted population was well specified in a large majority of studies with only two studies missing this information. A majority of studies applied to the Stroke population (N = 79), followed by CP population (N = 30) and SCI population (N = 15). The spasticity severity of the test group was provided in 108 studies.

#### Assessment methods comparison

In order to estimate which of the main assessment method categories (defined in the previous section) benefit from the more positive evaluation across the different psychometric properties, Table 3 reports the percentage of studies in each method, with what is considered a good psychometric property: a fully valid construct ($=2$), a Significant discriminant validity, a Strong or Very Strong correlation with clinical scales, an Excellent repeatability and any evaluation of the sensitivity.

**Table 3:** Summary of the studies in the literature, categorised into the Manual (M), Controlled (C), Voluntary (V) and Static (NS) approaches and type of measure

Stretch	Measure	Construct	Discriminant	Clinical	Repeatability	Sensitivity	# limbs	#	References
		(2/2)	(Significant)	($\ge$Strong)	(Excellent)	(Reported)	(P/PC+HC)		
M	MA+KI	65	4	27	19	8	453/45+22	26	[17, 40–64]
	MA+KI+FT	89	33	11	33	0	259/0+78	9	[38, 55, 65–71]
	KI+FT	13	63	13	13	0	344/16+29	8	[41, 72–78]
	KI	14	57	57	57	0	119/19+93	7	[35–37, 79–82]
	MA	0	33	17	17	0	242/41+40	6	[83–88]
	Other	33	33	0	0	0	26/0+8	3	[89–91]
C	KI+FT	36	36	23	18	5	791/75+411	22	[34, 92–112]
	MA+KI+FT	85	54	8	31	0	268/0+163	13	[113–125]
	MA+KI	67	0	0	0	0	115/8+22	6	[60, 126–130]
	KI	0	0	100	100	0	46/46+192	1	[131]
V	MA	0	20	20	0	0	60/22+8	5	[132–136]
	MA+KI	60	40	40	0	0	80/34+37	5	[59, 137–140]
	Other	0	33	33	0	0	80/80+124	3	[141–143]
NS	MP	0	83	50	0	0	257/153+0	6	[95, 95, 132, 144–146]
	MA	0	40	20	0	0	178/116+64	5	[70, 81, 146–148]
	Other	0	80	0	40	0	76/47+17	5	[39, 96, 122, 149, 150]

It shows the percentage of the studies in each category that showed a construct validity of 2 (out of 2), a Significant discriminant validity, a greater than Strong correlation with a clinical measure, an Excellent repeatability and the percentage of those that included a sensitivity evaluation. The table also reports the total sample size for each category as: number of tested limbs (P), own control limbs (PC) and healthy subjects’ control limbs (HC); and the number of studies in each of the category. Note that these results do not account for the individual sample size of each study and aggregate studies reporting a poor property and studies not reporting it

None of the different assessment methods demonstrate a good or even systematic validation across the five psychometric properties. Among the methods relying on Manual stretching, the ones using the larger set of measurements (M-MA+KI+FT) have an overall better validation. The simpler approach (M-KI), requiring the simpler equipment, has a good validation overall even if its construct validity remains low. Similarly, among methods relying on a Controlled stretching, the ones with the larger set of measurements (C-MA+KI+FT) demonstrate the best overall properties. Approaches relying on either Voluntary movements (V-) or on a static measurement (NS-) have a low construct validity score and generally suffer from an absence of repeatability evaluation.

#### Outcome measures comparison

The same analysis was performed based on the studies outcome measure(s). The results for the most commonly used measures are presented in Table 4.

**Table 4:** Summary of the studies in the literature evaluating the most common outcome measures (used in 10 or more studies)

Outcome measure	Construct	Discriminant	Clinical	Repeatability	Sensitivity	# limbs	#	References
	(2/2)	(Significant)	($\ge$Strong)	(Excellent)	(Reported)	(P/PC+HC)		
MA level	54	24	14	19	3	951/122+232	37	[17, 38, 40, 41, 49, 53, 55, 55, 58, 59, 59, 60, 60, 63–65, 67, 68, 70, 70, 71, 83–88, 91, 113, 118, 123, 127, 132, 134, 136, 137, 143]
Catch angle	35	6	6	47	0	364/50+250	17	[47, 49, 55, 57, 62, 63, 66–68, 71, 73, 78, 82, 114, 115, 118, 131]
FT (resistive)	47	41	18	24	0	458/9+236	17	[34, 38, 55, 67, 68, 70, 90, 93, 101–105, 112, 119, 123, 143]
FT (neural)	88	53	18	29	6	388/42+239	17	[70, 77, 80, 90, 94–99, 116–118, 120–122, 124]
TSRT	100	0	21	0	7	247/8+11	14	[42, 43, 46, 48, 50–52, 54, 61, 79, 126, 127, 129, 140]
EMG onset	62	15	15	0	0	268/0+85	13	[17, 38, 56, 60, 60, 63, 66, 119, 120, 124, 127, 130, 134]
Work	70	60	20	40	0	317/0+108	10	[38, 55, 67, 68, 71, 73, 77, 106, 119, 123]

It shows the percentage of the studies for each of the outcome measures that showed a construct validity of 2 (out of 2), a Significant discriminant validity, a greater than Strong correlation with a clinical measure, an Excellent repeatability and the percentage of those that included a sensitivity evaluation. The table also reports the total sample size for each outcome measure as: number of tested limbs (P), own control limbs (PC) and healthy subjects’ control limbs (HC); and the number of studies in each outcome measure. Note that these results do not account for the individual sample size of each study and aggregate studies reporting a poor property and studies not reporting it

**Fig. 3:**
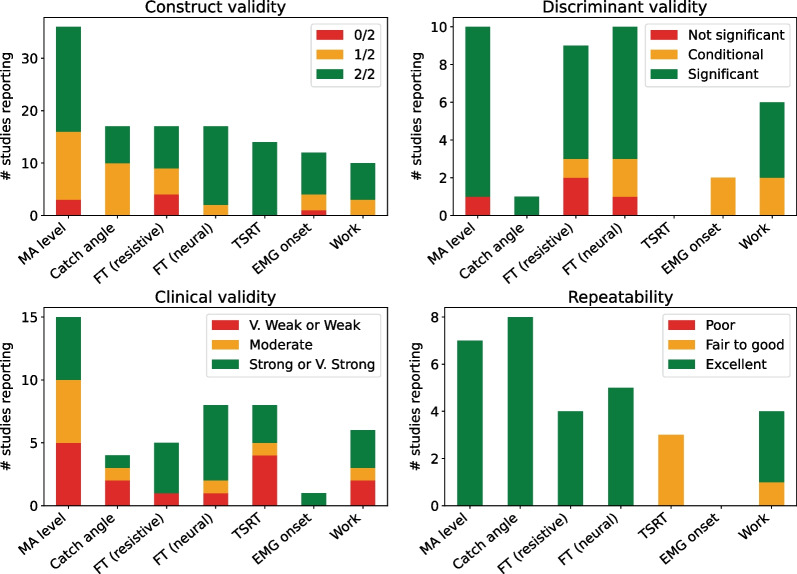
Response on the different psychometric properties for the more commonly adopted outcome measures (in 10 or more studies). Sensitivity being reported in only three studies is not presented. Note that this does account only for studies reporting the given property and as such leads to a very inequal total number across the different properties (and different vertical scales)

A more detailed analysis accounting only for studies reporting on a specific property is presented on Fig. 3. When evaluated, most outcomes demonstrate an Excellent repeatability and a positive discriminant validity. It is to note still, that the use of TSRT and the presence of EMG onset are very rarely evaluated along these properties. Overall, across the spectrum, only the Force/Torque measure, either as a whole (*i.e. *resistive) or isolating the neural component, and the Work measure demonstrate good properties in a majority of studies. This similar behaviour is not surprising, as these two outcome measures are relatively similar, the Work being the integration of the Force (or Torque) along the stretching movement.

Only one study reported properties of a good level across the first four psychometric properties (Additional file 1), and this for the MA level outcome measure when applied at the knee joint with subjects with CP [38]. The same authors, in a different study with the same population at the ankle, were the only ones to also report good construct validity, discriminant validity and repeatability (but without assessing clinical validity) [77]. This applied to the overall Work, specific Neural Work and Torque outcome measures.

### Usability analysis of valid approaches

Usability was evaluated and analysed for 54 studies which specifically evaluated spasticity and so had a construct validity of 2/2.

#### Usability comparison by method approaches

Less than half of the studies’ procedures (N = 19) could be administered in less than 10 mins, making them comparable to the MAS or MTS. It is of note that most of these 19 studies were in the Manual Stretch method category which generally required minimal time for equipment setup. In contrast, the majority of studies had an administration time as much as a typical intervention session (10–30 mins, N = 21) or an extended intervention session (30–60 mins, N = 14).

In terms of equipment cost, nearly half of the studies (N = 26) had a cost between $1000 and $10,000 USD. Meanwhile, six studies had a cost of $10,000–50,000 USD and 21 studies had a cost more than $50,000 USD, where most of these studies used a robotic device (REE or REXO) to perform Controlled stretching. A relatively low cost (less than $1000 USD) was only found in one study, which combined a musculoskeletal model and Kinematic measurements from three IMUs during Manual stretching to predict the velocity-dependent TSRT [79].

The portability analysis showed the assessment equipment was not movable in nearly half of the studies (N = 26). Only 11 studies used a device which could be transportable from room-to-room, and 17 studies (all in the Manual Stretch category) used easily transportable equipment.

#### Usability comparison by outcome measures

**Fig. 4:**
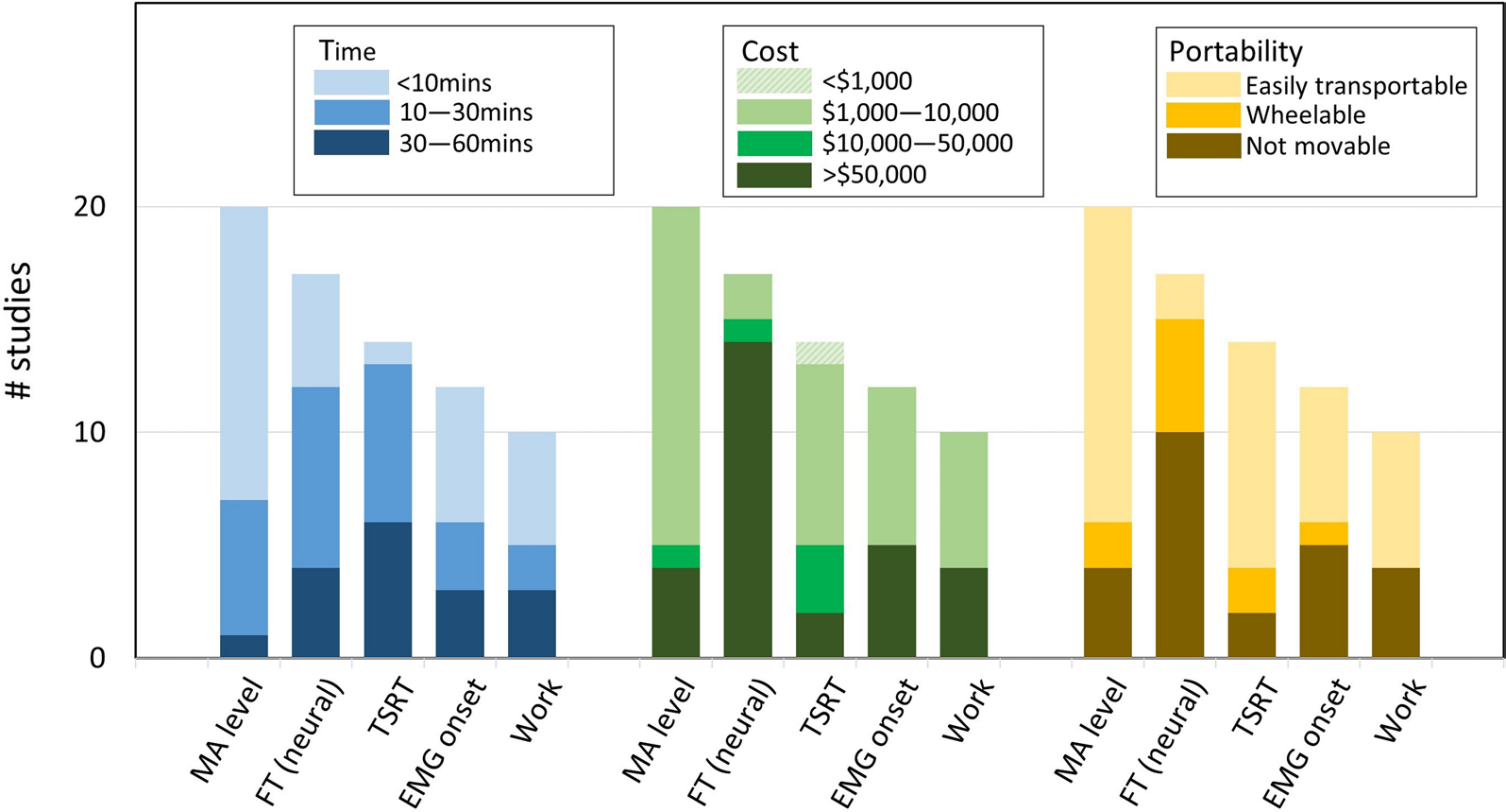
Usability analysis on the equipment cost, administration time and portability for the more commonly adopted outcome measures

In order to compare which validated assessment methods and their associated outcome measures have advantages in practicality, the usability for the most commonly used outcome measures (used in 10 or more studies) is presented in Fig. 4.

MA level approaches had an overall good usability across administration time, equipment cost and portability. More than half of the studies measuring MA level had an administration time of less than 10 mins, which is thus comparable to a MAS or MTS, they required an equipment costing between $1000 and $10,000 USD and scored high on portability.

Overall, the good usability of MA level relied on simple equipment used (e.g*. *EMG) and a relatively small number of Manual stretches in most of these studies.

Although TSRT demonstrated a similar performance on equipment cost and portability as MA level, its administration time was longer, more commonly of more than 30 mins as it requires a larger number of stretches. Indeed, a larger number of stretches is required to elicit sufficient Dynamic Strectch Reflex Thresholds (DSRT) data points to obtain a reliable TSRT value.

In contrast with MA level and TSRT, most FT (neural) studies had an equipment cost over $50,000 USD and lacked equipment movability as these assessment methods were generally performed on a robotic system (REE or REXO). Additionally, more than half of the FT (neural) studies required more than 10 mins for measurement.

## Discussion

The diversity of outcome measures shows that there is no clear agreement on efficient method for such assessment. The psychometric properties of the different measures are not well explored.

A very large spectrum of methods and outcome measures have been developed and used in studies with various methodology approaches. This development seems to be at the expense of limited formal evaluation of the proposed methods. Many studies neglect the evaluation of important psychometric properties of the measures. This is evident for the sensitivity (present in only three studies) and also repeatability which is not evaluated in a majority of studies. Clinical validity is more often present. If it appears important to provide a benchmark against accepted scales, its significance remains limited given the low specificity and inter/intra rater properties of the MAS and MTS (the most frequently encountered). This correlation cannot be considered alone to characterise an appropriate measure of spasticity. It would thus appear more appropriate to evaluate proposed methods against relatively well established ones such as Muscle Activity approaches (MA level) or ones properly isolating the stretch reflex in Controlled stretches (FT neural).

### Measures specificity to spasticity

The diversity of proposed methods and the limited construct validity (<2) in more than half the studies show the limited specificity of the methods evaluated. This is illustrated by the wide use of catch angles measures considered only at a single velocity, or the use of a MAS equivalent, also known to not be velocity-dependent. It was noted that, as suggested by McPherson et al. [4], catch angles may be used to construct a valid outcome measure (representing the stretch-reflex sensitivity) but only when their velocity dependence is considered. This lack of specificity in the literature is in agreement with a conclusion of a previous systematic review which found that a “majority of studies rely on methods that assess resistance to passive movement rather than spasticity” [23].

Still, the importance of specificity might be relative for clinical use. Typically, Botulinum toxin type A injection decisions can be made on the basis of static postures [151] or MAS scores [152] to address both hypertonicity and spasticity all-together. As such, when a specific measure is not required, it appears that simple instrumented methods relying on a manual stretching and a simple kinematic measurement [35–37, 79–82] could be favoured.

### Lack of repeatability and sensitivity evaluation

Overall, most outcome measures demonstrate an excellent repeatability when reported but with the notable exception of the TSRT and the presence of EMG onset (Fig. 3). This absence of repeatability evaluation is especially problematic given that these two measures are the ones with the higher construct validity overall—because they take advantage of a Muscle Activity measure—and are such very relevant approaches.

In general, repeatability, which is a fundamental property relatively straightforward to evaluate is very much lacking for most methods and outcome measures, and care should be taken to fill in this gap.

The sensitivity of the outcome measures is even more critically lacking from the literature, with only three studies proposing such evaluation. This confirms and extends a previous finding about robotic assessments of spasticity by van der Velden et al. [22]. The recommendation of the authors to invest more effort “in studying diagnostic accuracy” and its “added value for clinical care” can be extended to all existing instrumented measures.

### Usability and clinical implications

One of the main objectives of the different methodology developments in the literature is to provide alternatives to the MAS and MTS scales in clinical practice. These scales are criticised for their limited repeatability, specificity and sensitivity but have the major advantage of not requiring a specific equipment and being quick to administer with minimal training. As such, usability considerations are important when looking at possible alternatives.

A number of existing methods address this issue and have an estimated administration time of less than 10 minutes. Those mostly include instrumented Manual stretching methods measuring a Muscle Activity reaction and the Kinematics (e.g. [55]) or Force/Torque reaction (e.g. [77]). Lower cost alternatives relying only on a kinematic measure, provided by either a goniometer [80] or an IMU [35, 36, 79] have been proposed but have reported relatively poor psychometric properties, except for [79] and [80] (see Additional file 1).

It is also to note that, if TSRT is an interesting approach quite well explored, it can only be recommended as a comparison point in research studies as it requires a large number of stretches to be efficient, thus increasing its administration time.

Another, less specific alternatives to the MAS and MTS are static methods not relying on any stretching movements (NS-) and measuring either intrinsic Muscle Properties using Ultra-Sound [132, 144–146] or measuring the H-reflex using EMG and electrical stimulation [70, 81]. These approaches, past their low construct validity, have a good discriminant validity and a Moderate to Very Strong clinical validity, but no repeatability nor sensitivity evaluation.

Overall, Muscle Activity measures (using EMG) of Manual stretches seem to constitute the go-to alternative to existing clinical scales, given their short administration time but also relative low-cost (<$10,000 for most of them). These Manual methods tend to have a lower equipment cost than their Controlled counterparts which require a robotic system but this additional cost is often defrayed given that when robotic systems are used for spasticity assessment, this is generally not their only—or even primary—use, as discussed in [21]. In addition, Muscle Activity based methods require an appropriate placement of EMG electrodes which may require specific experience. The choice between Controlled-Torque methods and Manual-Muscle Activity one is thus still open depending on the equipment available and clinicians experience.

### Limitations

The diversity of outcome measures and variety of objectives of the studies make it difficult to draw specific conclusions. As such, one limitation of this review is the lack of analysis for every different joint and pathology. It is clear that practical considerations may not allow a straightforward translation of one method from one joint to another (e.g. sEMG placement or robotic devices fitting and adaption to the joints morphology) but such analysis would require a more narrow scope. Similarly regarding the different pathologies, the assessment needs, and limbs presentation, might vary slightly for the different pathologies and so affect each method differently.

In addition, the construct validity considered in this review intentionally does not characterise the physiological mechanisms of spasticity specifically: no distinction is made between methods estimating an increased sensitivity of the stretch reflex and methods estimating an increase of the reaction amplitude. This approach thus assumes that both effects may exist and can potentially characterise spasticity. This is expected to be aligned with current clinical definitions of spasticity.

The usability of the different methods in clinical practice is based on estimations of the equipment cost and administration time. It is acknowledged that the cost does not include the expertise that may be required by some methods and that the cost of the equipment itself can significantly vary. As such this remains only an approximation used for comparisons between methods. Similarly, the estimated administration time can highly depend on the patient presentation but also expertise of the assessor. It is only relevant here as a comparison between methods and against the commonly used clinical scales, MAS and MTS, which have the advantage of being fast to administer.

Finally, the choice to include conference proceedings within the scope appeared important given the importance of such publications in the engineering field which contributes to the development of the assessment methods. Nevertheless, this may have introduced a bias when analysing the validation of the methods, given that some preliminary publication may not provide a full validation, complemented in a different publication. This approach also tends to aggregate studies which aim to introduce new evaluation methods with ones focusing on a more careful analysis of the psychometric properties.

## Conclusions

The review found a large variety of technology assisted methods and associated outcome measures to assess spasticity. These methods generally lack systematic evaluation of their psychometric properties. It thus appears that some consolidation of knowledge around existing approaches is required and that no ready-to-use alternative to existing clinical scales (MAS and MTS) is yet fully validated. Nevertheless, methods measuring a Muscle Activity reaction to manual stretches appear as promising practical method to be investigated further. Similarly, and when robotic systems are readily available, measures relying on a specific Torque (or Work) reaction to a controlled stretching can also be recommended.

### Supplementary Information

**Additional  file 1: Table S1.** Assessment methods extraction table. 

**Additional file 2: Table S2.** Psychometric properties extraction table. 

## Data Availability

All data generated or analysed during this study are included in this published article and its Additional files.

## Appendix

**Table 5:** Definition of administration time used for usability evaluation

Action	Standard duration
EMG/MMG sensor placement	1 min per sensor
IMU sensor placement	1 min per sensor
Electrogoniometer placement	1 min per sensor
Dynamometer placement	1 min per sensor
Instrumented orthosis placement	1 min per device
Robotic end-effector adjusted and donned	5–10 mins per device
Robotic exoskeleton adjusted and donned	5–10 mins per device
Motion capture system calibration	10 mins per system
Limb stretching followed by a rest	0.5 mins per action
Stimulation followed by a rest	0.25 mins per action
Maximum voluntary contraction test	1 min per action

**Table 6:** Definition of equipment costs used for the usability evaluation

Equipment	Standard costing	Representative equipment
	(in USD)	
EMG/MMG sensor	$1500 per sensor	Delsys Trigno System
IMU sensor	$100 per sensor	WitMotion WT61C 6 Axis Sensor
Electrogoniometer	$1000 per sensor	Biosignalsplux Goniometer
Dynamometer	$1000 per sensor	Hoggan Scientific MicroFET2 Digital Dynamometer
Electrical stimulator	$300 per device	Ultra 9000 Multifunction Stimulator
Ultra-Sound system	$20,000 per device	Chison Sonobook 9 Color Doppler Ultrasound
Robotic end-effector	$10,000–100,000 per device	Custom 1 DoF device to Biodex Isokinetic System 4 Pro
Robotic exoskeleton	$10,000–100,000 per device	Custom 1 DoF device to Hocoma Armeo Power
Motion capture system	$100,000 per system	Vicon Motion Capture System
Instrumented orthosis	Summation of all mounted sensors costs	

## Abbreviations

MAS: Modified Ashworth Scale
MTS: Modified Tardieu Scale
EMG: ElectroMyoGraphy
sEMG: Surface ElectroMyoGraphy
IMU: Inertial Measurement Unit
US: Ultra-Sound
ICC: Intraclass Correlation Coefficient
TSRT: Tonic Stretch Reflex Threshold
CP: Cerebral Palsy
SCI: Spinal Cord Injury
KI: Kinematic
MA: Muscle Activity
FT: Force/Torque
MP: Muscle Property
M: Manual (stretch)
C: Controlled (stretch)
V: Voluntary (stretch)
NS: No Stretch
